# 

**Published:** 1880-08

**Authors:** 


### THE BUFFALO Medical and Surgical Journal.

INDEX MEDICUS.

EDITORS:

THOS. LOTHROP, M. D., A. R. DAVIDSON, M. D.,

P. W. VAN PEYMA, M. D., HERMAN MYNTER, M. D.,

LUCIEN HOWE, M. D.

### CONTENTS:

PAGE.

ORIGINAL COMMUNICATIONS:

Addison's Disease, by Thos. F. Rochester, M. D., . . . . . . . . . 1

CLINICAL REPORTS:

Urethral Chancroid, Cicatricial Stricture, by Henry P. Cooke, M. D., . . . . 8
Hospital Notes—Sloughing and Destruction of the Anus and Rectum, with Prolapse of the Rectum four inches—Relief by Operation. Service of Dr. C. C. F. Gay. 9
Ruptured Perineum, Reported by Dr. C. C. Frederick, . . . . . . . . 12

TRANSLATIONS:

Ligature of Left Carotid Artery for Hemorrhage following Kiotomy, by Hadar Liden, Med. Lic. Translated by Herman Mynter, M. D., . . . . . . . 13
The Micrococcus of Bluish Pus, by P. W. Van Peyma, M. D., . . . . . 14
Friction in Insomnia, by F. Peterson, M. D., 15
A Case of Erysipelatous Pneumonia, by F. Peterson, M. D., . . . . . . 16

SELECTIONS:

Digestive Ferments, by Wm. Roberts, M.D., 17
Etiology of Summer Diarrhea, . . . . 23
On the Influence of Maternal Shock in the Production of Fœtal Monstrosities, by Thomas Wilson, M.R.C.S., L.R.C.P., . 25
An Improved Method of Perineorrhaphy, by E. S. Bunker, M. D., . . . . . 30
Treatment of Sub-Involution of the Uterus, by James Braithwaite, M. D., . . 31
Suturing Divided Nerve Trunks, by Lewis S. Pilcher, M. D., . . . . 34
On Chancrous Folliculitis of the Vulva, or Follicular Soft Chancre, . . . . . 37
Contraction of Fingers, . . . . . 38
Tonga in Neuralgia, . . . . . . 38
Iron on Digestion, . . . . . . . 39
Phosphate of Lime, . . . . . . 39
Subcutaneous Injection of Fowler's Solution, 40
Bichloride of Mercury in Dysentery and Diarrhea, . . . . . . . 41
Boracite, . . . . . . . . 41
Eczema, . . . . . . . . . . 42

EDITORIAL:

The Buffalo Medical and Surgical Journal, . 42
The Buffalo State Asylum for the Insane, . 43
The Health Reports, . . . . . . 44

REVIEWS, . . . . . . . 45

